# HIV-1 re-suppression on a first-line regimen despite the presence of phenotypic drug resistance

**DOI:** 10.1371/journal.pone.0234937

**Published:** 2020-06-18

**Authors:** Adriaan E. Basson, Salome Charalambous, Christopher J. Hoffmann, Lynn Morris

**Affiliations:** 1 Centre for HIV and STIs, National Institute for Communicable Diseases of The National Health Laboratory Services, Johannesburg, Gauteng, South Africa; 2 School of Pathology, Faculty of Health Sciences, University of the Witwatersrand, Johannesburg, Gauteng, South Africa; 3 The Aurum Institute, Johannesburg, Gauteng, South Africa; 4 School of Public Health, Faculty of Health Sciences, University of The Witwatersrand, Johannesburg, Gauteng, South Africa; 5 Johns Hopkins University, School of Medicine, Baltimore, Maryland, United States of America; University of Pittsburgh, UNITED STATES

## Abstract

We have previously reported on HIV-1 infected patients who fail anti-retroviral therapy but manage to re-suppress without a regimen change despite harbouring major drug resistance mutations. Here we explore phenotypic drug resistance in such patients in order to better understand this phenomenon. Patients (n = 71) failing a non-nucleoside reverse transcriptase inhibitor (NNRTI)-based regimen, but who subsequently re-suppressed on the same regimen, were assessed for HIV-1 genotypic drug resistance through Sanger sequencing. A subset (n = 23) of these samples, as well as genotypically matched samples from patients who did not re-suppress (n = 19), were further assessed for phenotypic drug resistance in an *in vitro* single cycle assay. Half of the patients (n = 36/71, 51%) harboured genotypic drug resistance, with M184V (n = 18/36, 50%) and K103N (n = 16/36, 44%) being the most prevalent mutations. No significant difference in the median time to re-suppression (31–39 weeks) were observed for either group (p = 0.41). However, re-suppressors with mutant virus rebounded significantly earlier than those with wild-type virus (16 vs. 33 weeks; p = 0.014). Similar phenotypic drug resistance profiles were observed between patients who re-suppressed and patients who failed to re-suppress. While most remained susceptible to stavudine (d4T) and zidovudine (AZT), both groups showed a reduced susceptibility to 3TC and NNRTIs. HIV- 1 infected patients on an NNRTI-based regimen can achieve viral re-suppression on the same regimen despite harbouring viruses with genotypic and phenotypic drug resistance. However, re-suppression was less durable in those with resistance, reinforcing the importance of appropriate regimen choices, ongoing viral load monitoring and adherence counselling.

## Introduction

The use of antiretroviral therapy (ART) has a significant impact on the control of HIV-1 infection and HIV associated morbidity [1]. In eastern and southern Africa, home to more than 19 million people living with HIV, new HIV infections have declined by nearly a third between 2010 and 2017, with a 42% decline in AIDS-related deaths [2]. South Africa’s national anti-retroviral treatment (ART) program was rolled out in 2004 and is currently the largest treatment program in the world with ~4.3 million people receiving ART by 2017 [3].

The South African national guidelines for the management of HIV infection promote viral load testing for monitoring viral suppression on ART, as well as for diagnosing treatment failure [4]. At the time of this study, patients were initiated on an NNRTI-based first-line regimen and monitored by an annual viral load (VL) test. After two consecutive VL tests >1,000 copies/ml, patients were switched to a protease inhibitor (PI)-based second-line regimen. Patients failing an NNRTI-based first-line regimen with genotypic drug resistance mutations typically present with M184V/I, K65R, and/or thymidine analogue mutations (TAMs) and K103N, V106M/A and/or Y181C as the most prevalent NRTI and NNRTI mutations, respectively [5]. However, several studies have observed that 16%-71% of patients with viral breakthrough are able to re-suppress on the same NNRTI-based regimen after adherence intensification [6–13]. Re-suppression can be long-lasting and achievable for up to a median of 2.4 years [14].

Re-suppression has been observed in patients with major genotypic drug resistance mutations particularly those failing an NNRTI-based first-line regimen with M184V and K103N [7, 11, 13, 15]. The presence of such genotypic drug resistance mutations is usually associated with a decrease in the effectiveness of ART. However, the impact of these resistance mutations in the scenario of re-suppression is less clear. Here, we report on the genotypic resistance profiles of patients who re-suppressed on the same regimen, and performed *in vitro* phenotypic resistance testing to evaluate genotypic drug resistance in the context of re-suppression and failure.

## Materials and methods

### Study cohort

ART-naive patients were recruited and enrolled in a previously described workplace ART program within the mining industry, between November 2002 and May 2006, and initiated on an NNRTI-based combination ART regimen [13, 16]. At the time of initiation of ART, patients were offered the opportunity to participate in the evaluation cohort. Patients with CD4 count <250 cells/mm^3^; WHO stage 3 and CD4 count <350 cells/ mm^3^; or WHO stage 4, who initiated ART, were included in the overall cohort. CD4 counts and HIV RNA levels (VL) were determined before initiation, after 6 weeks on ART and every 6 months thereafter. Patients with a detectable viral load of >1,000 copies/ml after previously being undetectable, a sustained increase in viral load of >0.6 log from its lowest point and a return to 50% of the pre-treatment viral load were eligible to switch to a PI-based second line regimen. Due to concerns regarding adherence, tolerability of the second-line regimen, and premature regimen switching many patients where not switched to a second-line regimen until multiple elevated viral loads and, often, a notable CD4 count decline [17]. The patient demographics and study characteristics have been reported elsewhere [13]. Briefly, 93% (n = 3,479/3,727) of patients who met the inclusion criteria were male with a median age of 42 years and median CD4 count of 147 cells/mm3 at cART initiation. The median follow-up was 17.4 months, with 6,118 person-years of follow-up. The current study expands on previous studies from this cohort [13, 14] by including a larger number of samples for drug resistance genotyping, and is representative of the larger population of patients on NNRTI-based first-line ART regimens in South Africa. Patients with virologic failure and resuppression were included in this study based on the availability of stored serum for resistance testing. In addition, phenotypic drug resistance testing is performed on a subset of samples with a focus on resuppression and failure among individuals with genotypic HIV drug resistance. Ethics approvals were provided by the University of the Witwatersrand Human Research Ethics Committee and Johns Hopkins University, and participants provided written consent.

### Genotypic drug resistance analysis

Total nucleic acid was extracted from patient plasma on the MagNA Pure LC 2.0 instrument (Roche, Switzerland). Viral RNA was reverse transcribed with the ThermoScript^™^ RT-PCR system (Invitrogen^™^, USA) and a ~1,150 kb HIV-1 reverse transcriptase (RT) fragment (from amino acid 82 in PR, to amino acid 367 in RT), amplified by nested PCR using the Expand High-Fidelity^PLUS^ PCR System (Roche, Switzerland). The second-round primers introduced HpaI endonuclease restrictions sites on either side of the amplicons, as required for vector construction in the phenotypic assay [18]. Amplicons were sequenced by population-based sequencing using the ABI BigDye^®^ Terminator V3.1 Cycle Sequencing Kit and the ABI Prism 3130xl Genetic Analyser (Applied Biosystems, USA). The Stanford HIV Drug Resistance Algorithm was used for quality assessment of sequences and mutation recognition [19].

### Phenotypic drug susceptibility analysis

The *in vitro* phenotypic drug susceptibilities of a subset of samples, containing viral variants with various genotypic drug resistance patterns, were assessed in a single-cycle non-replicative assay as previously described [18, 20]. Briefly, patient-derived pseudoviruses were constructed by ligating HpaI restricted nested PCR products into the p8.MJ4ΔRT HIV-1 subtype C expression vector. The patient-derived vectors included the RT connection/RNaseH domains to incorporate mutations (e.g. N348I [21]) that may impact on ARV drug susceptibility. The sequences of the inserts of the resulting clones were confirmed by population-based sequencing as described above. Clones whose genotypes matched the corresponding amplicons sequences were used. When genotypes did not match, more than one clone was used for that patient to ensure that all mutations were included. Pseudoviruses were generated by co-transfection of the patient-specific clones with vectors pM.DG and pCSFLW into HEK293T cells, and harvesting virus-containing supernatants 48 hours later. HEK293T cells were obtained from the American Type Culture Collection (Catalogue number CRL-3216) through Duke University School of Medicine. This cell line was not verified by our laboratory.

For the phenotypic analysis of patient-derived pseudoviruses, serial dilutions of anti-retroviral drugs were prepared in complete DMEM in flat-bottom 96-well culture plates. After the addition of HEK293T cells and pseudovirus, the plates were incubated for 48 hours and viral activity quantified through the expression of firefly luciferase using the Bright-Glo luciferase assay substrate (Promega, USA) on a Victor3 multi-label reader (PerkinElmer, USA). The p8.MJ4 expression vector was used as a wild-type control. Virus inocula were standardized to produce a luminescence of 1×10^4^ to 1×10^5^ relative light units (RLU) after 48 h of incubation with HEK293T cells in drug-free medium. The fold-change (FC) difference in inhibitory concentration-50 (IC_50_) relative to the wild-type control was calculated for each patient-derived pseudovirus. The lower technical cut-off (TCO) for each drug was determined using the 99th percentile of the average IC_50_ for the wild-type pseudovirus, assessed in two or more independent screens of each drug. The TCOs were as follows: AZT (2.1FC); d4T (2.9FC); ABC (1.1FC); 3TC (2.7FC); FTC (1.5FC); TDF (2.0FC); EFV (2.9FC); NVP (2.0FC). Samples with FC values at or below the TCO for a particular drug were classified as being susceptible, while those with FC values above the TCO were classified as having a reduced susceptibility. The TCO cut-off values used in this analysis were not linked to clinical correlates or outcomes but were used merely to rank the responses for the ARVs used in our assay.

### Drug level testing of EFV and NVP

Plasma samples from patients with resistance mutations who re-suppressed (n = 19) or failed (n = 16) were submitted to the National Health Laboratory Services (NHLS) for drug level testing by ultra-performance liquid chromatography–tandem quadruple mass spectrometry (UPLC-MS/MS) using a Waters Acquity UPLC T3 Column. For each sample, the protein in 25 μl of plasma was precipitated with 200 μl acetonitrile and 5 μl of deuterated internal standard. After centrifugation for 10 minutes at 14,000 x g, 4 μl of the precipitate was analysed using a gradient of dH_2_O-acetonitrile (9:1) containing 0.1% formic acid. The limits of detection (LOD) for 3TC, EFV and NVP were 50.9 ng/ml, 285.7 ng/ml and 15.4 ng/ml, respectively.

### Calculations and statistical analysis

For the comparative analysis of patients characteristics between different groups, the unpaired t-test with Welch’s correction factor (two-tailed) and contingency analyses with Fisher’s exact test (two-sided, confidence interval (CI): 95%) were performed using GraphPad Prism version 5.08 for Windows (GraphPad Software, San Diego California USA, www.graphpad.com). For the *in vitro* phenotypic assay, IC_50_ and FC values were determined using Microsoft Excel 2010 (Microsoft, Redmond, Washington, USA).

## Results

### Genotypic drug resistance analysis of patients who re-suppressed

For this study, 71 patients who showed an initial viral suppression (VL<400), followed by a viral breakthrough and a subsequent viral re-suppression on the same regimen were selected. The majority of patients were on a d4T/3TC (53/71 [75%]) or AZT/3TC (15/71 [21%]) containing regimen receiving either EFV (41/68) or NVP (27/68) The remaining 4% (3/71) of patients were on a regimen of TDF/FTC/EFV, TDF/FTC/NVP or ABC/3TC/EFV, respectively.

Almost all patients (70/71 [99%]) were infected with HIV-1 subtype C. One patient was infected with an HIV-1 CRF02_AG recombinant strain. Genotypic resistance analysis indicated that half of the patients (36/71 [51%]) contained virus with major genotypic drug resistance mutations (Table 1): NNRTI mutations (34/36 [94%]), NRTI mutations (18/36 [50%]) or both (16/36 [44%]). The most prevalent NNRTI mutations were K103N (16/36 [44%]) and V106M (9/36 [25%]). The prevalence of other major NNRTI resistance mutations (Y181CS, Y188CH, G190A and M230L) was ≤8%. The most predominant NRTI mutation was M184V (18/36 [50%]). Thymidine analogue mutations (TAMs) were present in four patients (11%), receiving either d4T (2/4), AZT (1/4) or ABC (1/4) (S1 Table). A quarter (9/36 [25%]) of the samples contained both M184V and K103N mutations.

**Table 1:** Characteristics of 71 patients who re-suppressed with or without genotypic resistance.

Re-suppressor patient characteristics	^a^Wild-type virus n(%)	Mutant virus n(%)	
**Total**	**35**	**36**	
d4T/3TC + EFV	18 (51)	15 (42)	
d4T/3TC + NVP	12 (34)	8 (22)	
AZT/3TC + EFV	2 (6)	6 (17)	
AZT/3TC + NVP	2 (6)	5 (14)	
TDF/FTC + EFV	-	1 (3)	
TDF/FTC + NVP	1 (3)	-	
ABC/3TC + EFV	-	1 (3)	
**Genotypes**		**36**	
**All NNRTI**		**34 (94)**	
K103N		16 (44)	
V106M		9 (25)	
Y181CS		3 (8)	
Y188H		3 (8)	
G190A		1 (3)	
M230L		1 (3)	
**All NRTI**		**18 (50)**	
M184V		18 (50)	
TAMs		4 (11)	
**All NRTI + All NNRTI**		**16 (44)**	
K103N+M184V		9 (25)	
**Viral load (log**_**10**_ **copies/ml)**	*median (IQR)*	*median (IQR)*	*p-value*
During full monitoring period	1.39 (1.78)	2.31 (3.31)	0.005 **
At sampling point	4.35 (4.96)	3.89 (4.29)	0.017 *
**Type of suppression after sampling point**	**35**	**36**	
Full suppression	27 (77)	23 (64)	
Transient viremia	1 (3)	2 (6)	
Repeat failure	7 (20)	11 (31)	
**Time (weeks)**	*median (IQR)*	*median (IQR)*	*p-value*
Full monitoring period	157 (61)	178 (80)	0.020 *
Between visits	26 (4)	26 (8)	0.508 ns
At sampling point (breakthrough)	113 (90)	125 (75)	0.047 *
Re-suppressed	39 (56)	31 (45)	0.408 ns
To repeat failure	33 (32)	16 (13)	0.014 *

IQR, Inter-Quartile Range; SD, Standard Deviation; ns, not significant;

* significant;

** very significant.

^a^Three patients (PID337454, PID120314 and PID316947) with polymorphisms (A98S, K103R, E138S, V179I) in the absence of major mutations were classified as having a wild-type genotype.

Patients with mutant virus had a significantly (p = 0.005) higher median VL (2.31 log_10_ copies/ml; IQR 1.39–3.32) during the full monitoring period compared to patients with wild-type virus (1.39 log_10_ copies/ml; IQR 1.39–1.93). However, at the sampling time point, viral loads were significantly (p = 0.017) lower among those with mutant viruses (3.89 log_10_ copies/ml vs. 4.35 log_10_ copies/ml). Treatment responses after the sampling point showed that patients with mutant viruses had lower rates of virological re-suppression (to VL<400: 23 [64%]), higher rates of transient viremia (VL 400–1,000: 2 [6%]) and more repeat failures (VL>1,000: 11 [31%]) than those with wild-type virus (27 [77%]; 1 [3%]; 7 [20%], respectively).

Patients with mutant viruses were monitored for significantly (p = 0.020) longer periods (178 weeks, IQR 156–237) than those with wild-type virus (157 weeks, IQR 133–193). No significant difference (p = 0.508) was observed in the median time between visits for those with wild-type (26 weeks; IQR 24–29) and mutant virus (26 weeks; IQR 21–29). Sampling time points were significantly (p = 0.047) later for those with mutant virus (125 weeks; IQR 89–164) than for those with wild-type virus (113 weeks; IQR 52–142). No significant difference (p = 0.408) was observed in the median duration of re-suppression until repeat failure between those with wild-type (39 weeks; IQR 21–76) and mutant (31 weeks; IQR 25–63) virus. The maximum time of re-suppression was 116 and 98 weeks for wild-type and mutant samples, respectively (not shown). For patients with a subsequent repeat failure, a significant difference (p = 0.014) was observed in time to the repeat failure for those with wild-type (median 33 weeks; IQR 24–57) and mutant (median 16 weeks; IQR 13–26) virus.

### Phenotypic drug resistance analysis of patients who re-suppressed or failed

From the 36 patients who re-supressed on the same regimen in the presence of one or more major NRTI and/or NNRTI mutations, 21 patients with various degrees of genotypic drug resistance (i.e. single, double and more complex combinations of major drug resistance mutations) representative of the cohort were selected for further phenotypic analysis. As a comparator group, 18 patients with similar drug resistance mutation profiles, who did not re-suppress or who had an in-class drug switch before re-suppression, were included (termed failing patients). For both groups, genotypic resistance patterns varied with some patients having single mutations and others with more complex combinations of up to 9 mutations (S2 Table). Two re-suppressor patients and one failure patient with wild-type genotypes were included as phenotypically susceptible controls.

When comparing the re-suppressor subgroup (n = 21) to the failing patients (n = 18), both groups had a median time between HIV RNA assays of ~24 weeks (IQR 20–27 weeks) and were followed over a similar time period of 193–243 weeks (Table 2). However, samples from failing patients were obtained at significantly (p = 0.006) later median time points (177 weeks; IQR 130–233 weeks) than for those who re-suppressed (153 weeks; IQR 100–163 weeks). Failing patients had significantly (p = 0.033) higher median viral loads (VL = 3.40 log_10_ copies/ml; IQR 2.98–3.98) during the entire monitoring period than those who re-suppressed (VL = 2.85 log_10_ copies/ml; IQR 1.99–3.4). Higher median viral loads were significantly associated with failure (OR 4.67, 95% CI 1.14–19.08, p = 0.049). There was no significant difference (p = 0.944) in the median viral loads at the sampling points. A significantly (p<0.0001) higher number of patients who failed (n = 17, 94%) had breakthrough events before the sampling point, compared to patients who re-suppressed (n = 4, 19%). We found that the number of previous breakthrough events were significantly predictive of failure (OR = 72.25, 95% CI 7.30–715.4, p<0.0001). Both groups of patients were on similar regimens and displayed similar genotypic resistance profiles, with the K103N (≥57%) and M184V (≥71%) mutations being the most prevalent.

**Table 2:** Patients with genotypic resistance who re-suppressed compared to those who failed.

Patient characteristics	Re-suppressed n(%)	Failed n(%)	
**Time (weeks)**	*median (IQR)*	*median (IQR)*	*p-value*
Total follow-up period	193 (79)	243 (120)	0.172 ns
Between visits	25 (8)	24 (6)	0.272 ns
At sampling point	153 (64)	177 (103)	0.006 **
**Viral load (log**_**10**_ **copies/ml)**			
During follow-up	2.85 (3.39)	3.40 (3.93)	0.033 *
At sampling point	3.83 (4.37)	4.05 (4.56)	0.944 ns
**Breakthrough events**			
Before sampling	n = 4/21 (19%)	n = 17/18 (94%)	<0.0001 ***
**Total**	**21**	**18**	
d4T/3TC + EFV	5 (24)	4 (22)	
d4T/3TC + NVP	6 (29)	8 (44)	
AZT/3TC + EFV	5 (24)	5 (28)	
AZT/3TC + NVP	4 (19)	1 (6)	
TDF/FTC + EFV	1 (5)	-	
**Genotype**	**21**	**18**	
**All NNRTI**	**20 (95)**	**18 (100)**	
K103N	12 (57)	11 (61)	
V106M	4 (19)	5 (28)	
Y181CS	2 (10)	1 (6)	
Y188CHL	3 (14)	3 (17)	
G190A	1 (5)	2 (11)	
M230L	1 (5)	1 (6)	
**All NRTI**	**15 (71)**	**14 (78)**	
M184V	15 (71)	14 (78)	
TAMs	4 (19)	5 (28)	
K65R	1 (5)	1 (6)	
**K103N+M184V**	**9 (43)**	**7 (39)**	

IQR, Inter-Quartile Range; SD, Standard Deviation; ns, not significant;

* significant;

** very significant;

*** extremely significant

**Fig 1:**
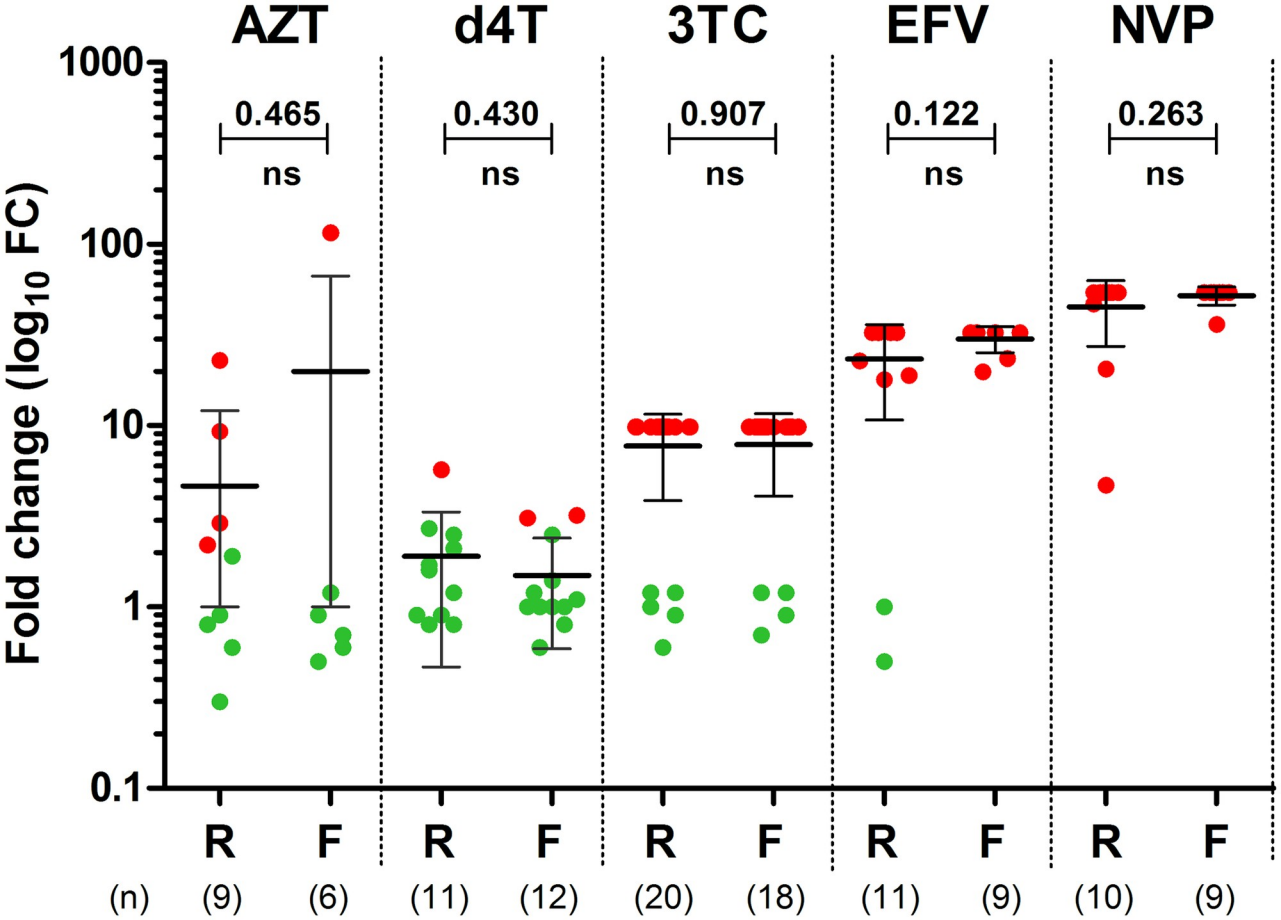
*In vitro* phenotypic drug susceptibility of pseudoviruses from re-suppressing and failing patients. The fold change (FC) in drug susceptibility of pseudoviruses from re-suppressing and failing patients for each antiretroviral drug used in their ARV regimens. The number of patients in each group is indicated below the x-axis. Averages, standard deviations (error bars) and statistical significances (p-values) are displayed. The cut-off for each drug is: AZT (FC<2.1), d4T (FC <2.9), 3TC (FC <2.7), EFV (2.9) and NVP (2.0). Pseudoviruses that are susceptible are shown in green and those with reduced susceptibility in red. ns–not significant. R: Patients who re-suppressed, F: Patients who failed.

A total of 43 pseudoviruses were generated from both groups and tested for *in vitro* phenotypic drug susceptibility against the ARVs that matched the regimen of the corresponding patients (S2 Table). The majority of cloned RT fragments had a genotype that agreed with their corresponding amplicon; 3 samples (PID486741, PID433732 and PID067414) had clones that showed genotypic variation and more than one clone was used in these cases. When comparing the fold-change in phenotypic susceptibility for each drug between the patients who re-suppressed or failed (Fig 1), the average FC values between the two groups of patients were generally comparable and not significantly different (p≥0.122). Although the average fold change for AZT was higher in patients who failed (FC = 19.9, SD 45.8) than for patients who re-suppressed (FC = 4.6, SD 7.4) this was not significantly different (p = 0.465). Almost all pseudoviruses (41/43 [95%]) showed high-level reductions in susceptibility to EFV or NVP (FC 4.7–54.1). Those with M184V mutation (33/43 [77%]) had a reduced susceptibility to 3TC (FC = 9.8, the limit of the assay), while those without M184V were fully susceptible (10/43 [23%], FC≤1.5) (S2 Table). A minority of pseudoviruses were resistant to d4T (3/23 [13%]) or AZT (5/15 [33%]). Three wild-type pseudoviruses were included as controls and were fully susceptible to all drugs. A subset of patient plasma samples were screened for detectable levels of 3TC, EFV and NVP as a proxy for ART compliance. A total of 19 and 16 samples from patients with genotypic resistance who re-suppressed or failed were available for drug level testing, respectively. A total of 63% (12/19) and 69% (11/16) of re-suppressor and failure patients, respectively, had detectable drug levels and this was not predictive of either re-suppressing or failing (OR 0.779, 95% CI 0.190–3.19, p = 1.000) (S2 Table).

**Fig 2:**
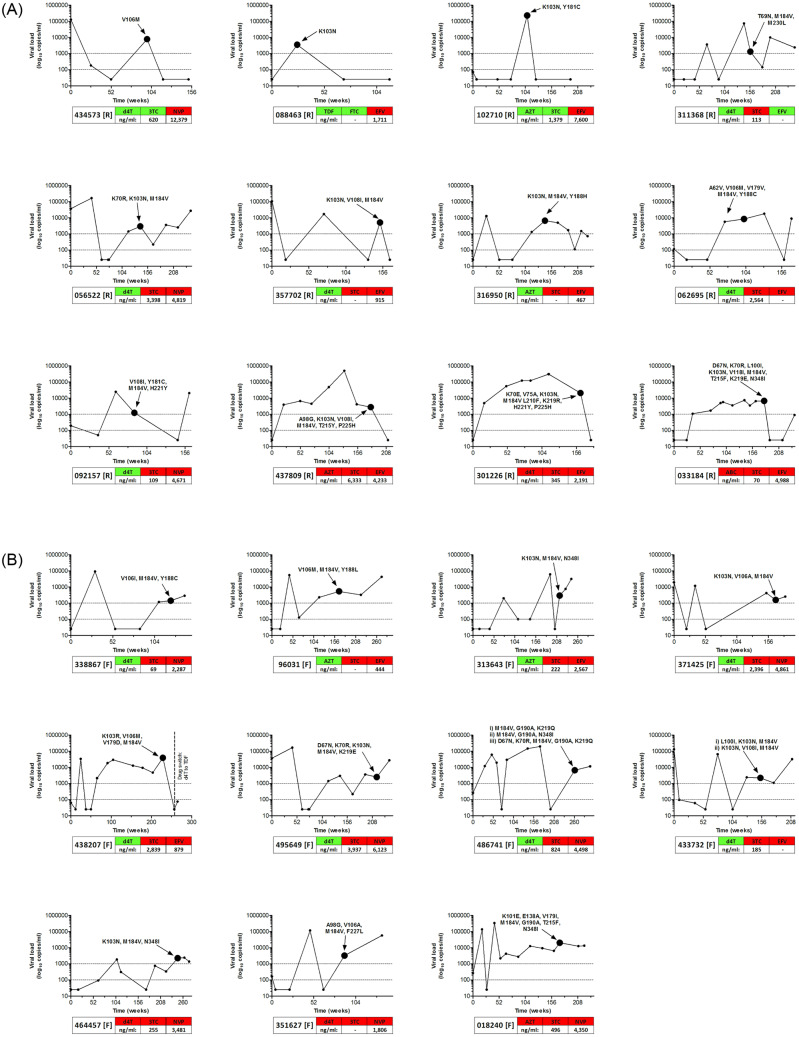
Characteristics of patients with detectable drug levels, who re-suppressed or failed. The viral loads of re-suppressing (A) and failing (B) patients are displayed over the monitoring period (in weeks). Sampling time points (large dot) and genotypic drug resistance mutations are shown. Drug regimens and *in vitro* phenotypic responses are indicated, with green indicating full susceptibility and red indicating a reduced susceptibility. Detectable drug levels are shown in ng/ml. Viral loads of 100 copies/ml and 1,000 copies/ml are indicated by dotted lines on the y-axis.

The longitudinal profiles of patients with detectable drug levels are shown in Fig 2, together with their major drug resistance mutations and phenotypic susceptibilities. All 12 resuppressor patients showed control of viremia after the breakthrough event. Some patients had single mutations, mostly to NNRTIs while others had multiple mutations against both NNRTIs and NRTIs. There was a good correlation between the presence of genotypic resistance and an increase in fold-change to the corresponding ARV. This was observed despite the presence of detectable drug levels in all patients. Similar profiles were seen among the 11 failing patients except that viral levels remained high (>1,000) after the breakthrough event. One patient (PID 438207) re-suppressed but only after an in-class drug switch. Thus, despite the similarities in detectable drug levels, the presence of genotypic drug resistance mutations and a reduced phenotypic susceptibility to one or more antiretroviral drugs in their regimen, some were able to resuppress while other patients failed treatment.

## Discussion

It is not uncommon for patients on ART that experience virological failure to re-suppress on the same regimen despite the presence of major drug resistance mutations that accumulated during sub-optimal ART. Here, we examined the impact of drug resistance mutations on the phenotypic response to ARVs in such patients from a workplace HIV programme that was initiated before roll-out of the national ARV treatment program in South Africa [13].

We found that half of the patients that re-suppressed after virologic breakthrough contained major NRTI (e.g. M184V) and NNRTI (e.g. K103N, V106M) drug resistance mutations, typically observed in patients failing an NNRTI-based regimen [22, 23]. These patients had more frequent repeat failures and lower viral loads at breakthrough events than patients that re-suppressed with wild-type virus. Overall, however, re-suppressors with drug resistance mutations had significantly higher viral loads during the treatment monitoring period. Although the median duration of re-suppression was similar between patients with or without genotypic resistance mutations (~7–9 months), those with resistance mutations were likely to have a repeat viral rebound much sooner, within a median of 17 weeks after a suppressed VL. An *in vitro* phenotypic drug susceptibility analysis between these patients and a comparator group of failing patients showed similar, predictable phenotypic resistance patterns. The majority of patients in both groups showed a decreased susceptibility to two antiretroviral drugs (3TC and EFVor NVP) while remaining fully or partially susceptible to only one antiretroviral drug (d4T or AZT). We found that the phenotypic response to ARVs was not predictable of failure, but that the number of previous viral breakthroughs were. These findings suggest that adherence, rather than viral susceptibility to a sub-optimal ART regimen, may be the determining factor with regards to re-suppression and failure.

It is well described that non-adherence is linked to the development of drug resistance and HIV disease progression [24]. Furthermore, a delayed antiretroviral switch is associated with an increased risk for the development of drug resistance, opportunistic infection (OI) and death [25, 26], and an increased risk of the transmission of drug resistant virus [27]. The relationship between adherence and the probability of drug resistance development differs dramatically across drug class [28, 29]. Resistance to NNRTI-based regimens occurs at low to moderate levels of adherence. Thus, in the case of re-suppressors with wild-type virus, it is likely that complete abstinence from cART occurred. In the case of re-suppressors with genotypic drug resistance mutations, low to intermediate adherence to the EFV/NVP-based regimen would have selected for the drug resistance mutations observed [28]. Despite the presence of genotypic resistance mutations, these patients were still able to re-suppress and we have shown no significant difference in phenotypic drug susceptibilities compared to patients who failed. For both the re-suppressor and failure groups, sensitivity to d4T or AZT remained, while loss in sensitivity to 3TC and EFV/NVP occurred. Although resistance to EFV/NVP is fairly absolute, NRTIs such as 3TC and AZT may have sustained ARV activity despite long intervals of partially suppressive therapy and the presence of resistant HIV-1 [30]. In addition, the M184V resistance mutation selected for by ABC, FTC or 3TC imparts a clinical benefit due to reduced viral fitness, increased RT fidelity, and hypersensitization to other NRTIs among others [31–33]. Despite this, however, drug resistance mutations selected for during sub-optimal ART may impact on future drug options and drug efficacies [34]. This is particularly relevant to NNRTI mutations and cross-resistance to newer second-generation NNRTIs (i.e. Etravirine and Rilpivirine) [18].

The detection of early virological failure should provide an opportunity to increase adherence counselling and repeat viral load testing before a switch in treatment [6]. Enhanced adherence counselling can result in the resuppression of a substantial number of patients failing cART and avoid unnecessary drug regimen switches [35]. Viral load monitoring has proven to be an important tool in monitoring and reinforcing adherence [8]. Same-day point-of-care viral load testing has shown to be useful in identifying patients who require immediate adherence councelling [36]. The inclusion of biomarkers and self-reported adherence could produce a potentially more accurate measure of adherence to cART [37]. Although therapeutic drug monitoring (TDM) can be useful in certain clinical settings, it should be done with caution. In the context of adherence monitoring, serum drug concentrations only reflect drug administration within a short time period prior to blood collection, and do not provide information on sustained drug dosing [38]. This is particularly true for drugs with short half-lives. Furthermore, ARV compliance in the days immediately before blood collection but lack of compliance otherwise (so-called “white coat syndrome”) [39] could provide misleading conclusions in regard to adherence.

Non-adherence is multifactorial and solutions to circumvent this are complex [40]. Efforts should be intensified to identify patients at risk of poor adherence, and establish the support that is needed to overcome the most important barriers to adherence [41]. Enhanced adherence counselling alone, however, may not provide sufficient additional support than the current standard of care [42] and several successful interventions have been used to improve adherence and viral suppression [43, 44]. This includes regimen simplification [45], use of single-tablet regimens [46], short message service (SMS) text messages [47], and community-based ART programs [48, 49]. Although adherence intensification is likely to increase the probability of re-suppression on the same regimen, this does not always hold true [42, 50, 51]. Resistance testing, where feasible, has been proposed for those with higher levels of viremia at failure to improve discernment between patients in need of a switch to second-line therapy versus those in need of adherence support interventions [51]. Although genotypic drug resistance testing is not typically performed in low-to-middle income countries due to cost implications, alternative assays (e.g. point mutation assays) may provide a feasible option in the latter scenario [52]. Also, risk score algorithms may be useful in low-to-middle income countries to expedite drug or regimen switching for patients in need of more effective ART regimens [53].

There are some limitations in the current study. Firstly, only a limited number of patient samples were selected for phenotypic drug resistance testing, thereby potentially underpowering and limiting generalization of the results. Secondly, only a limited number of TDF-treated patients were included in this study and in light of the high prevalence of the K65R mutation in sub-Saharan African countries [54, 55], it is unclear whether our conclusions are applicable to patients re-suppressing or failing TDF-based regimens in this setting. Thirdly, in the absence of biological cut-off values for our *in vitro* phenotypic assay, an assumption was made that FC values above the TCO values translated into a decrease in drug susceptibility. This could potentially have lead to the under- or over-estimation of phenotypic drug resistance in the patient samples. Lastly, although we used drug level testing as a surrugate for adherence, no reliable adherence data was available for use in this study and could potentially limit the conclusions that were drawn. In addition, CD4+ T-cell counts and pre-treatment viral loads are important factors that could contribute to viral failure [56, 57] but were not considered in this study.

In conclusion, the ability of patients to re-suppress on the same regimen is possible despite genotypic resistance mutations and a reduced phenotypic susceptibility to most of the drugs in the regimen. However, the accumulation of additional genotypic drug resistance mutations in patients on sub-optimal ART regimens may have implication for future treatment options. Adherence is fundamentally important for a durable and sustained suppressive cART regimen. Monitoring and support is pivotal and should be provided sooner than later. Drug resistance testing may be beneficial in guiding the management of virologic failure for some individuals in resource-limited settings.

## Supporting information

S1 Table Characteristics and genotypic drug resistance mutations of re-suppressor patients. (XLSX)

S2 Table Characteristics, genotypic- and phenotypic drug resistance of a subset of resuppressing and failing patients. (XLSX)

## References

[1] Montaner JS, Hogg R, Wood E, Kerr T, Tyndall M, Levy AR, et al The case for expanding access to highly active antiretroviral therapy to curb the growth of the HIV epidemic. Lancet (London, England). 2006;368(9534):531–6. Epub 2006/08/08. 10.1016/s0140-6736(06)69162-9 . 16890841

[2] UNAIDS. UNAIDS 2018 Reference. UNAIDS/JC2929E. 2018.

[3] UNAIDS. UNAIDS ‘AIDSinfo’. UNAIDS "AIDSinfo'. 2018.

[4] NDOH. National Consolidated Guidelines for the Prevention of Mother-to-Child Transmission of HIV (pMTCT) and the Management of HIV in Children, Adolescents and Adults. 2015.

[5] Steegen K, Bronze M, Papathanasopoulos MA, van Zyl G, Goedhals D, Variava E, et al HIV-1 antiretroviral drug resistance patterns in patients failing NNRTI-based treatment: results from a national survey in South Africa. J Antimicrob Chemother. 2017;72(1):210–9. 10.1093/jac/dkw358 . 27659733

[6] Shet A, Neogi U, Kumarasamy N, DeCosta A, Shastri S, Rewari BB. Virological efficacy with first-line antiretroviral treatment in India: predictors of viral failure and evidence of viral resuppression. Trop Med Int Health. 2015;20(11):1462–72. Epub 2015/07/07. 10.1111/tmi.12563 . 26146863

[7] Gupta RK, Goodall RL, Ranopa M, Kityo C, Munderi P, Lyagoba F, et al High rate of HIV resuppression after viral failure on first-line antiretroviral therapy in the absence of switch to second-line therapy. Clin Infect Dis. 2014;58(7):1023–6. Epub 2013/12/20. 10.1093/cid/cit933 24352348 PMC3952602

[8] Bonner K, Mezochow A, Roberts T, Ford N, Cohn J. Viral load monitoring as a tool to reinforce adherence: a systematic review. Journal of acquired immune deficiency syndromes (1999). 2013;64(1):74–8. Epub 2013/06/19. 10.1097/QAI.0b013e31829f05ac . 23774877

[9] Johnston V, Fielding KL, Charalambous S, Churchyard G, Phillips A, Grant AD. Outcomes following virological failure and predictors of switching to second-line antiretroviral therapy in a South African treatment program. Journal of acquired immune deficiency syndromes (1999). 2012;61(3):370–80. Epub 2012/07/24. 10.1097/QAI.0b013e318266ee3f 22820803 PMC3840925

[10] Barth RE, Aitken SC, Tempelman H, Geelen SP, van Bussel EM, Hoepelman AI, et al Accumulation of drug resistance and loss of therapeutic options precede commonly used criteria for treatment failure in HIV-1 subtype-C-infected patients. Antiviral therapy. 2012;17(2):377–86. Epub 2012/02/03. 10.3851/IMP2010 . 22297391

[11] El-Khatib Z, Delong AK, Katzenstein D, Ekstrom AM, Ledwaba J, Mohapi L, et al Drug resistance patterns and virus re-suppression among HIV-1 subtype C infected patients receiving non-nucleoside reverse transcriptase inhibitors in South Africa. Journal of AIDS & clinical research. 2011;2(117). Epub 2011/09/20. 10.4172/2155-6113.1000117 21927716 PMC3174802

[12] Orrell C, Kaplan R, Wood R, Bekker LG. Virological breakthrough: a risk factor for loss to followup in a large community-based cohort on antiretroviral therapy. AIDS research and treatment. 2011;2011:469127 Epub 2011/07/01. 10.1155/2011/469127 21716724 PMC3119419

[13] Hoffmann CJ, Charalambous S, Sim J, Ledwaba J, Schwikkard G, Chaisson RE, et al Viremia, resuppression, and time to resistance in human immunodeficiency virus (HIV) subtype C during first-line antiretroviral therapy in South Africa. Clin Infect Dis. 2009;49(12):1928–35. 10.1086/648444 19911963 PMC2789416

[14] Hoffmann CJ, Charalambous S, Grant AD, Morris L, Churchyard GJ, Chaisson RE. Durable HIV RNA resuppression after virologic failure while remaining on a first-line regimen: a cohort study. Trop Med Int Health. 2014;19(2):236–9. 10.1111/tmi.12237 24588012 PMC4070658

[15] Fox Z, Phillips A, Cohen C, Neuhaus J, Baxter J, Emery S, et al Viral resuppression and detection of drug resistance following interruption of a suppressive non-nucleoside reverse transcriptase inhibitor-based regimen. Aids. 2008;22(17):2279–89. Epub 2008/11/05. 10.1097/QAD.0b013e328311d16f 18981767 PMC2745911

[16] Charalambous S, Grant AD, Day JH, Pemba L, Chaisson RE, Kruger P, et al Establishing a workplace antiretroviral therapy programme in South Africa. AIDS care. 2007;19(1):34–41. 10.1080/09500340600677872 . 17129856

[17] Hoffmann CJ, Maritz J, van Zyl GU. CD4 count-based failure criteria combined with viral load monitoring may trigger worse switch decisions than viral load monitoring alone. Trop Med Int Health. 2016;21(2):219–23. 10.1111/tmi.12639 . 26584666 PMC6717435

[18] Basson AE, Rhee SY, Parry CM, El-Khatib Z, Charalambous S, De Oliveira T, et al Impact of drug resistance-associated amino acid changes in HIV-1 subtype C on susceptibility to newer nonnucleoside reverse transcriptase inhibitors. Antimicrob Agents Chemother. 2015;59(2):960–71. 10.1128/AAC.04215-14 25421485 PMC4335849

[19] Liu TF, Shafer RW. Web resources for HIV type 1 genotypic-resistance test interpretation. Clin Infect Dis. 2006;42(11):1608–18. 10.1086/503914 16652319 PMC2547473

[20] Parry CM, Kohli A, Boinett CJ, Towers GJ, McCormick AL, Pillay D. Gag determinants of fitness and drug susceptibility in protease inhibitor-resistant human immunodeficiency virus type 1. J Virol. 2009;83(18):9094–101. 10.1128/JVI.02356-08 19587031 PMC2738216

[21] McCormick AL, Parry CM, Crombe A, Goodall RL, Gupta RK, Kaleebu P, et al Impact of the N348I mutation in HIV-1 reverse transcriptase on nonnucleoside reverse transcriptase inhibitor resistance in non-subtype B HIV-1. Antimicrob Agents Chemother. 2011;55(4):1806–9. 10.1128/AAC.01197-10 21282419 PMC3067126

[22] Barth RE, van der Loeff MF, Schuurman R, Hoepelman AI, Wensing AM. Virological follow-up of adult patients in antiretroviral treatment programmes in sub-Saharan Africa: a systematic review. The Lancet Infectious diseases. 2010;10(3):155–66. Epub 2010/02/27. 10.1016/S1473-3099(09)70328-7 . 20185094

[23] Huang A, Hogan JW, Luo X, DeLong A, Saravanan S, Wu Y, et al Global Comparison of Drug Resistance Mutations After First-Line Antiretroviral Therapy Across Human Immunodeficiency Virus-1 Subtypes. Open Forum Infect Dis. 2016;3(2):ofv158 10.1093/ofid/ofv158 27419147 PMC4943563

[24] Lucas GM. Antiretroviral adherence, drug resistance, viral fitness and HIV disease progression: a tangled web is woven. J Antimicrob Chemother. 2005;55(4):413–6. 10.1093/jac/dki042 . 15722389

[25] Murphy RA, Court R, Maartens G, Sunpath H. Second-Line Antiretroviral Therapy in Sub-Saharan Africa: It Is Time to Mind the Gaps. AIDS research and human retroviruses. 2017;33(12):1181–4. 10.1089/AID.2017.0134 28793781 PMC5709698

[26] Petersen ML, Tran L, Geng EH, Reynolds SJ, Kambugu A, Wood R, et al Delayed switch of antiretroviral therapy after virologic failure associated with elevated mortality among HIV-infected adults in Africa. AIDS. 2014;28(14):2097–107. 10.1097/QAD.0000000000000349 24977440 PMC4317283

[27] Ssempijja V, Nakigozi G, Chang L, Gray R, Wawer M, Ndyanabo A, et al Rates of switching to second-line antiretroviral therapy and impact of delayed switching on immunologic, virologic, and mortality outcomes among HIV-infected adults with virologic failure in Rakai, Uganda. BMC Infect Dis. 2017;17(1):582 10.1186/s12879-017-2680-6 28830382 PMC5568262

[28] Bangsberg DR, Moss AR, Deeks SG. Paradoxes of adherence and drug resistance to HIV antiretroviral therapy. J Antimicrob Chemother. 2004;53(5):696–9. 10.1093/jac/dkh162 . 15044425

[29] Rosenbloom DI, Hill AL, Rabi SA, Siliciano RF, Nowak MA. Antiretroviral dynamics determines HIV evolution and predicts therapy outcome. Nature medicine. 2012;18(9):1378–85. Epub 2012/09/04. 10.1038/nm.2892 22941277 PMC3490032

[30] Eron JJ Jr., Bartlett JA, Santana JL, Bellos NC, Johnson J, Keller A, et al Persistent antiretroviral activity of nucleoside analogues after prolonged zidovudine and lamivudine therapy as demonstrated by rapid loss of activity after discontinuation. Journal of acquired immune deficiency syndromes (1999). 2004;37(5):1581–3. Epub 2004/12/04. . 15577413 10.1097/00126334-200412150-00009

[31] Wainberg MA. Increased fidelity of drug-selected M184V mutated HIV-1 reverse transcriptase as the basis for the effectiveness of 3TC in HIV clinical trials. Leukemia. 1997;11 Suppl 3:85–8. Epub 1997/04/01. . 9209307

[32] Wainberg MA, Hsu M, Gu Z, Borkow G, Parniak MA. Effectiveness of 3TC in HIV clinical trials may be due in part to the M184V substitution in 3TC-resistant HIV-1 reverse transcriptase. Aids. 1996;10 Suppl 5:S3–10. Epub 1996/12/01. 10.1097/00002030-199612005-00002 . 9030390

[33] Turner D, Brenner BG, Routy JP, Petrella M, Wainberg MA. Rationale for maintenance of the M184v resistance mutation in human immunodeficiency virus type 1 reverse transcriptase in treatment experienced patients. The new microbiologica. 2004;27(2 Suppl 1):31–9. Epub 2005/01/14. . 15646062

[34] von Wyl V, Klimkait T, Yerly S, Nicca D, Furrer H, Cavassini M, et al Adherence as a predictor of the development of class-specific resistance mutations: the Swiss HIV Cohort Study. PloS one. 2013;8(10):e77691 10.1371/journal.pone.0077691 24147057 PMC3797701

[35] Ford N, Orrell C, Shubber Z, Apollo T, Vojnov L. HIV viral resuppression following an elevated viral load: a systematic review and meta-analysis. J Int AIDS Soc. 2019;22(11):e25415 10.1002/jia2.25415 31746541 PMC6864498

[36] Villa G, Abdullahi A, Owusu D, Smith C, Azumah M, Sayeed L, et al Determining virological suppression and resuppression by point-of-care viral load testing in a HIV care setting in sub-Saharan Africa. EClinicalMedicine. 2020;18:100231 10.1016/j.eclinm.2019.12.001 31922120 PMC6948257

[37] Rhead R, Masimirembwa C, Cooke G, Takaruza A, Nyamukapa C, Mutsimhi C, et al Might ART Adherence Estimates Be Improved by Combining Biomarker and Self-Report Data? PloS one. 2016;11(12):e0167852 Epub 2016/12/16. 10.1371/journal.pone.0167852 27973550 PMC5156419

[38] Gerber JG, Acosta EP. Therapeutic drug monitoring in the treatment of HIV-infection. J Clin Virol. 2003;27(2):117–28. 10.1016/s1386-6532(03)00033-7 . 12829033

[39] Pretorius E, Klinker H, Rosenkranz B. The role of therapeutic drug monitoring in the management of patients with human immunodeficiency virus infection. Ther Drug Monit. 2011;33(3):265–74. 10.1097/FTD.0b013e31821b42d1 . 21566505

[40] Iacob SA, Iacob DG, Jugulete G. Improving the Adherence to Antiretroviral Therapy, a Difficult but Essential Task for a Successful HIV Treatment-Clinical Points of View and Practical Considerations. Front Pharmacol. 2017;8:831 10.3389/fphar.2017.00831 29218008 PMC5703840

[41] Shubber Z, Mills EJ, Nachega JB, Vreeman R, Freitas M, Bock P, et al Patient-Reported Barriers to Adherence to Antiretroviral Therapy: A Systematic Review and Meta-Analysis. PLoS medicine. 2016;13(11):e1002183 Epub 2016/11/30. 10.1371/journal.pmed.1002183 27898679 PMC5127502

[42] Fox MP, Pascoe SJS, Huber AN, Murphy J, Phokojoe M, Gorgens M, et al Effectiveness of interventions for unstable patients on antiretroviral therapy in South Africa: results of a cluster-randomised evaluation. Trop Med Int Health. 2018;23(12):1314–25. 10.1111/tmi.13152 . 30281882

[43] Kanters S, Park JJ, Chan K, Socias ME, Ford N, Forrest JI, et al Interventions to improve adherence to antiretroviral therapy: a systematic review and network meta-analysis. The lancet HIV. 2017;4(1):e31–e40. Epub 2016/11/20. 10.1016/S2352-3018(16)30206-5 . 27863996

[44] Haberer JE, Sabin L, Amico KR, Orrell C, Galarraga O, Tsai AC, et al Improving antiretroviral therapy adherence in resource-limited settings at scale: a discussion of interventions and recommendations. J Int AIDS Soc. 2017;20(1):21371 10.7448/IAS.20.1.21371 28630651 PMC5467606

[45] Chen Y, Chen K, Kalichman SC. Barriers to HIV Medication Adherence as a Function of Regimen Simplification. Annals of behavioral medicine: a publication of the Society of Behavioral Medicine. 2016 Epub 2016/08/12. 10.1007/s12160-016-9827-3 . 27510955 PMC5253105

[46] Sutton SS, Ahuja D, Magagnoli J. What is the effect of pill burden on adherence to HIV antiretroviral therapy? JAAPA: official journal of the American Academy of Physician Assistants. 2016;29(11):16–7. Epub 2016/10/28. 10.1097/01.JAA.0000502870.89587.b6 . 27787272

[47] Georgette N, Siedner MJ, Zanoni B, Sibaya T, Petty CR, Carpenter S, et al The Acceptability and Perceived Usefulness of a Weekly Clinical SMS Program to Promote HIV Antiretroviral Medication Adherence in KwaZulu-Natal, South Africa. AIDS and behavior. 2016;20(11):2629–38. Epub 2016/10/21. 10.1007/s10461-016-1287-z 26781866 PMC4949151

[48] Mukherjee JS, Barry D, Weatherford RD, Desai IK, Farmer PE. Community-Based ART Programs: Sustaining Adherence and Follow-up. Current HIV/AIDS reports. 2016;13(6):359–66. Epub 2016/10/16. 10.1007/s11904-016-0335-7 27739018 PMC5106499

[49] Nachega JB, Adetokunboh O, Uthman OA, Knowlton AW, Altice FL, Schechter M, et al Community-Based Interventions to Improve and Sustain Antiretroviral Therapy Adherence, Retention in HIV Care and Clinical Outcomes in Low- and Middle-Income Countries for Achieving the UNAIDS 90-90-90 Targets. Current HIV/AIDS reports. 2016;13(5):241–55. Epub 2016/08/01. 10.1007/s11904-016-0325-9 . 27475643 PMC5357578

[50] Jobanputra K, Parker LA, Azih C, Okello V, Maphalala G, Kershberger B, et al Factors associated with virological failure and suppression after enhanced adherence counselling, in children, adolescents and adults on antiretroviral therapy for HIV in Swaziland. PloS one. 2015;10(2):e0116144 10.1371/journal.pone.0116144 25695494 PMC4335028

[51] McCluskey SM, Boum Y 2nd, Musinguzi N, Haberer JE, Martin JN, Hunt PW, et al Brief Report: Appraising Viral Load Thresholds and Adherence Support Recommendations in the World Health Organization Guidelines for Detection and Management of Virologic Failure. Journal of acquired immune deficiency syndromes (1999). 2017;76(2):183–7. 10.1097/QAI.0000000000001479 28628529 PMC5597473

[52] Rhee SY, Jordan MR, Raizes E, Chua A, Parkin N, Kantor R, et al HIV-1 Drug Resistance Mutations: Potential Applications for Point-of-Care Genotypic Resistance Testing. PloS one. 2015;10(12):e0145772 10.1371/journal.pone.0145772 26717411 PMC4696791

[53] Rutstein SE, Hosseinipour MC, Weinberger M, Wheeler SB, Biddle AK, Wallis CL, et al Predicting resistance as indicator for need to switch from first-line antiretroviral therapy among patients with elevated viral loads: development of a risk score algorithm. BMC Infect Dis. 2016;16:280 10.1186/s12879-016-1611-2 27296625 PMC4906700

[54] Skhosana L, Steegen K, Bronze M, Lukhwareni A, Letsoalo E, Papathanasopoulos MA, et al High prevalence of the K65R mutation in HIV-1 subtype C infected patients failing tenofovir-based first-line regimens in South Africa. PloS one. 2015;10(2):e0118145 10.1371/journal.pone.0118145 25659108 PMC4320083

[55] TenoRes Study G. Global epidemiology of drug resistance after failure of WHO recommended first-line regimens for adult HIV-1 infection: a multicentre retrospective cohort study. The Lancet Infectious diseases. 2016;16(5):565–75. 10.1016/S1473-3099(15)00536-8 26831472 PMC4835583

[56] Ceccherini-Silberstein F, Cozzi Lepri A, Alteri C, Merlini E, Surdo M, Marchetti G, et al Pre-ART HIV-1 DNA in CD4+ T cells correlates with baseline viro-immunological status and outcome in patients under first-line ART. J Antimicrob Chemother. 2018;73(12):3460–70. 10.1093/jac/dky350 . 30247724

[57] Fatti G, Grimwood A, Nachega JB, Nelson JA, LaSorda K, van Zyl G, et al Better Virological Outcomes Among People Living With Human Immunodeficiency Virus (HIV) Initiating Early Antiretroviral Treatment (CD4 Counts >/ = 500 Cells/microL) in the HIV Prevention Trials Network 071 (PopART) Trial in South Africa. Clin Infect Dis. 2020;70(3):395–403. 10.1093/cid/ciz214 . 30877753 PMC7768744

