# Supplementary material for: HIV-1 re-suppression on a first-line regimen despite the presence of phenotypic drug resistance
Source: PLoS One. 2020 Jun 18;15(6):e0234937. doi: 10.1371/journal.pone.0234937 (PMC7302689; doi:10.1371/journal.pone.0234937)
Supplement: Attachment — Submitted filename: Response to Reviewers.docx [file pone.0234937.s003.docx]

Phillip v Tobias Health Sciences Building 29 Princess of Wales Terrance (cnr York Road) Parktown 2193; Johannesburg; South Africa T +27 11 717 2103

[Maria.Papathanasopoulos@wits.ac.za](mailto:Maria.Papathanasopoulos@wits.ac.za)

Cristian Apetrei, MD, PhD

Academic Editor

PLOS ONE

13 May 2020

Dear Academic Editor:

We thank you and the Reviewer for devoting your time and effort in reviewing our manuscript entitled: “HIV-1 re-suppression on a first-line regimen despite the presence of phenotypic drug resistance” by Adriaan E Basson, Salome Charalambous, Christopher J Hoffmann and Lynn Morris, to be considered for publication as a research article in PLOS One. Please see below for our response to the points raised by yourself and the Reviewer.

1. Please ensure that your manuscript meets PLOS ONE's style requirements, including those for file naming.

Response: We have corrected the style of our manuscript to meet PLOS ONE’s requirements. This can be observed by referring to the track changes in the “Revised Manuscript with Track Changes”. The major changes related to converting the tables to cell-based Microsoft Excel worksheet objects.

2. In your Methods section, please provide additional details regarding the cell lines used in your study. Please include the source from which you obtained the cells, whether the cell line was verified, and if so, how it was verified.

Response: We have added the relevant information:

Page 7, lines 130 – 132: “HEK293T cells were obtained from the American Type Culture Collection (Catalogue number CRL-3216) through Duke University School of Medicine. This cell line was not verified by our laboratory.”

3. To comply with PLOS ONE submission guidelines, in your Methods section, please provide additional information regarding your statistical analyses. Specifically, please indicate which of the described statistical analyses were performed for which experiment.

Response: We have now clarified the use of statistical tests as follows:

Page 6, Lines 166 - 171: “**For the comparative analysis of patients characteristics between different groups,** the unpaired t-test with Welch’s correction factor (two-tailed) and contingency analyses **with Fisher’s exact test (two-sided, confidence interval (CI): 95%)** were performed using GraphPad Prism version 5.08 for Windows (GraphPad Software, San Diego California USA, www.graphpad.com). **For the *in vitro* phenotypic assay**, IC50 and FC values were **determined** using Microsoft Excel 2010 (Microsoft, Redmond, Washington, USA).”

The following sentence was removed since it relates to an analysis that were not included in this manuscript: “Logistic regression analysis was performed with NCSS 11 statistical software (NCSS, LLC. Kaysville, Utah, USA, ncss.com/software/ncss, 2016).”

4. Thank you for your ethics statement "ethics approvals were provided by the University of the Witwatersrand Human Research Ethics Committee and Johns Hopkins University, and participants provided written consent."

Response: The information has now been added.

Pages 5 - 6 , lines 95 – 101: “Ethics approvals were provided by the University of the Witwatersrand Human Research Ethics Committee and Johns Hopkins University, and participants provided written consent.”

5. In your Methods section, please provide additional information about the participant recruitment method and the demographic details of your participants. Please ensure you have provided sufficient details to replicate the analyses such as:
a) the recruitment date range (month and year),

Response: Recruitment occurred over the same time period as study enrolment. We have modified the text to reflect that:

Page 5, lines 74 – 76: “ART-naive patients were **recruited and enrolled** in a previously described workplace ART program within the mining industry, **between November 2002 and May 2006**, and initiated on an NNRTI-based combination ART regimen [13, 16].”

b) a description of any inclusion/exclusion criteria that were applied to participant recruitment,

Response: We have now included inclusion criteria:

Page 5, lines 78 – 79: “Patients with CD4 count <250 cells/mm3; WHO stage 3 and CD4 count <350 cells/ mm3; or WHO stage 4, who initiated ART, were included in this study.”

and

Page 5, lines 94 – 95: “Patients with virologic failure and resuppression were included in this study based on the availability of stored serum for resistance testing.”

c) a table of relevant demographic details,

Response: The patient demographics and study characteristics have previously been published elsewhere. However, we have made a reference to that publication in the text and added a very brief overview:

Page 5, lines 87 – 91: “The patient demographics and study characteristics have been reported elsewhere [13]. Briefly, 93% (n=3,479/3,727) of patients who met the inclusion criteria were male with a median age of 42 years and median CD4 count of 147 cells/mm3 at cART initiation. The median follow-up was 17.4 months, with 6118 person-years of follow-up.”

d) a statement as to whether your sample can be considered representative of a larger population, and

Response: We have added this to the text:

Page 5, lines 91 – 94: “The **current** study expands on previous studies from this cohort[13, 14] by including a larger number of samples for drug resistance genotyping, **and is representative of the larger population of patients on NNRTI-based first-line ART regimens in South Africa**.”

e) a description of how participants were recruited.

Response: We have added the information as follows:

Page 5, lines 76 – 78: “At the time of initiation of ART, patients were offered the opportunity to participate in the evaluation cohort."

6. We note that you have included the phrase “data not shown” in your manuscript.

“No significant difference (p>0.400) was observed in the total number of resistance mutations, NRTI mutations, TAMs or NNRTI mutations per sample between the two groups (data not shown).”

Unfortunately, this does not meet our data sharing requirements. PLOS does not permit references to inaccessible data.

Response: After careful consideration, we have decided to remove this sentence from the manuscript as Table 2 sufficiently illustrates similarity between the two groups of patients regarding regimens and genotypic drug resistance mutations.

7. Reviewer 1: Minor Comment

In lines 239 and 240, the authors have selected 21 patients for phenotypic analysis out of the 36 patients who were re-suppressed on the same regimen in the presence one of more major NRTI and/or NNRTI mutations. The authors have not explained why all the 36 patients were not considered for the phenotypic analysis or what criteria was used to select the 21 patients. Please elaborate.

Response: We have now clarified this in the text:

Page 12, lines 262 – 266: “From the 36 patients who re-supressed on the same regimen in the presence of one or more major NRTI and/or NNRTI mutations, 21 patients **with various degrees of genotypic drug resistance (i.e. single, double and more complex combinations of major drug resistance mutations)** **representative of the cohort** were selected for further phenotypic analysis.”

We hope that we have adequately addressed the issues that were raise, and that the manuscript is now suitable for publication in your journal.

Sincerely,

Adriaan E Basson

HIV Pathogenesis Research Unit

Department of Molecular Medicine and Haematology

University of the Witwatersrand

Johannesburg, South Africa

Tel./Fax: +2711-717-2581

Email: [adriaan.basson@wits.ac.za](mailto:adriaan.basson@wits.ac.za)
